# Multivariate brain-behaviour associations in psychiatric disorders

**DOI:** 10.1038/s41398-024-02954-4

**Published:** 2024-06-01

**Authors:** S. Vieira, T. A. W. Bolton, M. Schöttner, L. Baecker, A. Marquand, A. Mechelli, P. Hagmann

**Affiliations:** 1 Department of Radiology, Lausanne University Hospital and University of Lausanne, Lausanne, Switzerland; 2 Department of Psychosis Studies, Institute of Psychiatry, Psychology and Neuroscience, King’s College London, London, UK; 3 Center for Research in Neuropsychology and Cognitive Behavioral Intervention, Faculty of Psychology and Educational Sciences, University of Coimbra, Coimbra, Portugal; 4 Neurosurgery Service and Gamma Knife Center, Lausanne University Hospital, Lausanne, Switzerland; 5 Donders Institute for Brain, Cognition and Behavior, Radboud University Nijmegen, Nijmegen, The Netherlands; 6 Department for Cognitive Neuroscience, Radboud University Medical Center Nijmegen, Nijmegen, The Netherlands; 7 Department of Neuroimaging, Institute of Psychiatry, Psychology, & Neuroscience, King’s College London, London, UK

**Keywords:** Psychiatric disorders, Human behaviour

## Abstract

Mapping brain-behaviour associations is paramount to understand and treat psychiatric disorders. Standard approaches involve investigating the association between one brain and one behavioural variable (univariate) or multiple variables against one brain/behaviour feature (‘single’ multivariate). Recently, large multimodal datasets have propelled a new wave of studies that leverage on ‘doubly’ multivariate approaches capable of parsing the multifaceted nature of both brain and behaviour simultaneously. Within this movement, canonical correlation analysis (CCA) and partial least squares (PLS) emerge as the most popular techniques. Both seek to capture shared information between brain and behaviour in the form of latent variables. We provide an overview of these methods, review the literature in psychiatric disorders, and discuss the main challenges from a predictive modelling perspective. We identified 39 studies across four diagnostic groups: attention deficit and hyperactive disorder (ADHD, k = 4, N = 569), autism spectrum disorders (ASD, k = 6, N = 1731), major depressive disorder (MDD, k = 5, N = 938), psychosis spectrum disorders (PSD, k = 13, N = 1150) and one transdiagnostic group (TD, k = 11, N = 5731). Most studies (67%) used CCA and focused on the association between either brain morphology, resting-state functional connectivity or fractional anisotropy against symptoms and/or cognition. There were three main findings. First, most diagnoses shared a link between clinical/cognitive symptoms and two brain measures, namely frontal morphology/brain activity and white matter association fibres (tracts between cortical areas in the same hemisphere). Second, typically less investigated behavioural variables in multivariate models such as physical health (e.g., BMI, drug use) and clinical history (e.g., childhood trauma) were identified as important features. Finally, most studies were at risk of bias due to low sample size/feature ratio and/or in-sample testing only. We highlight the importance of carefully mitigating these sources of bias with an exemplar application of CCA.

## Introduction

Mapping the association between brain and behaviour has been a focus of psychiatric research for many decades. However, the analytical methods to achieve this have changed substantially over time. Historically, mass-univariate analysis, where many single brain features are linked to a single behavioural phenotype, have dominated the literature [1–4]. However, there is growing concern about the reproducibility of these findings, especially when carried out in typically small samples [5, 6]. This approach also does not take into account the mutual dependencies between different brain regions, nor is it consistent with the current view that behaviour is best explained by distributed neural networks, rather than localised regions [7–10]. Therefore, multivariate methods are better suited to capture brain–behaviour relationships. Within this context, two main families of methods are becoming increasingly popular: 1) mapping multiple brain features to one behavioural feature (i.e., ‘many-to-one’ associations) and 2) discovering latent brain-behaviour associations from multiple brain and multiple behaviour features (i.e., ‘many-to-many’ associations or ‘doubly multivariate’ methods). The former has enjoyed considerable interest in the last two decades, with a wealth of studies investigating how neural features can predict a range of univariate psychiatric outcomes such as functioning [11, 12], diagnosis [13–15] and response to treatment [16, 17] using popular approaches such as support vector machine (SVM) [18–20]. Within the second group, there are many approaches that could be used in principle, such as independent component analysis (ICA) and its variants (e.g., parallel ICA, joint ICA or linked ICA) [21], multilevel clustering [22], canonical correlation analysis (CCA) [23] and partial least squares [24] (PLS). The latter two emerge as the most established and popular techniques in brain-behaviour studies, as evidenced by several recent studies in the general population [25] and tutorials tailored to brain-behaviour investigations [26, 27]. There are many variations of CCA and PLS [27]. The two most used types of PLS in neuroimaging are Partial Least Squares Correlation (PLSC) and Partial Least Squares Regression (PLSR). PLSC is a correlational technique that estimates associations between two sets of data (e.g., behaviour and brain morphology), while PLSR is a regression technique that predicts one set of data from another (e.g., predicts behaviour from brain activity). Multiset CCA (mCCA) and mCCA+ICA are variations of CCA and have mostly been used to investigate the association between different imaging modalities or incorporate a behavioural constraint (use behavioural data to guide the fusion between imaging modalities) [28–31]. Here we focus on standard CCA and PLSC (henceforth PLS) (and their regularised variations) as they are the most commonly used approaches to investigate brain-behaviour associations [25, 32]. Both seek to capture shared information, in the form of latent variables, between two sets of multivariate data. Early applications in neuroimaging aimed at combining different imaging modalities, a process also known as multimodal fusion [33]. More recently, the same approach is being used to map brain and behaviour associations. This is achieved by attributing weights to the brain and behavioural variables such that their linear combination maximises the correlation (CCA) or covariance (PLS) between the resulting latent variables (see Box 1 for an overview; for a more in-depth explanation see Tabachnick et al., [34]). The main advantage of this approach is that the multivariate nature of both brain and behaviour can be modelled simultaneously. Indeed, although the standard use of ‘many-to-one’ approaches in psychiatric imaging acknowledges the multifaceted nature of brain data, they do not take into account that different aspects of behaviour also interact with each other [35]. Therefore, approaches such as CCA and PLS are better suited to honour the complex brain-behaviour associations by allowing to uncover joint multivariate relationships.

Although CCA/PLS were introduced many decades ago, they are ´data-hungry’ and can be computationally demanding. The release of large publicly available imaging datasets with comprehensive behavioural assessments, such as the Adolescent Brain Cognitive Development [36], Human Connectome Project [37] and UK BioBank [38], has propelled a renewed interest in the use of these methods to investigate brain-behaviour associations [39–43]. For example, specific brain patterns have been linked to perinatal and early life events, sociocognition, and urbanicity [44–46], different types of psychopathology [47], as well as physical features, lifestyle and cognition [48]. Notably, these methods may be particularly useful in psychiatry. Mental health disorders are best understood as a complex pattern of both neural and behavioural changes. For example, psychosis is characterised by widespread changes in brain morphology, structure and connectivity [49–51], but also by a pattern of positive, negative, mood and cognitive symptoms, insight and functioning, all relevant for diagnosis and treatment [52]. However, despite the consensus of the multivariate brain-behaviour interaction in psychiatric disorders, these studies remain rare; multimodal data is expensive and requires expertise in several data modalities (e.g., neuroimaging, cognition, psychopathology) as well as in the analytical methods to integrate them. Parallel to this, there is also a growing interest in investigating the generalisability and stability of brain-behaviour associations in psychiatry [27, 53, 54]. This is partly in response to the replication crisis in psychiatry/psychology [25] combined with the pursuit of translational clinical tools [55]. In practice, this means that the typical descriptive analytical framework, where the aim is to find above-chance associations in-sample, is being challenged and more emphasis is being given to predictive approaches, where the goal is to test whether a brain-behaviour association is stable and can be generalised to new data [56, 57]. The aim of this systematic review is to summarise the applications of commonly used ‘doubly’ multivariate methods, namely CCA and PLS, to investigate brain-behaviour associations in psychiatric disorders within this context, as well as discuss main challenges and future directions.

Box 1 Overview of canonical correlation analysis and partial least squares CCA and PLS are data-driven techniques that can extract latent associations between a set of multivariate brain data and a set of multivariate behavioural data. The former can comprise, for example, functional connectivity for selected brain regions, while the latter may include, *e.g*., information about symptoms, cognition, medication, duration of illness and psychiatric history. Simply put, CCA and PLS quantify the association between brain and behaviour by finding the weights, for each dataset, that maximise the link between the two.

Formally, consider brain and behaviour data matrices **X** and **Y**, of respective sizes *S* × *n*_*x*_ and *S* × *n*_*y*_, with *S* the number of subjects with available measurements and *n*_*x*_/*n*_*y*_ the number of brain/behavioural measures at hand. CCA and PLS extract associated weight vectors **w**_**x**_ and **w**_**y**_, of respective sizes *n*_*x*_ × 1 and *n*_*y*_ × 1. The products **L**_**x**_ = **X**·**w**_**x**_ and **L**_**y**_ = **Y**·**w**_**y**_ are, respectively, weighted combinations of the brain and behavioural measures for each subject; they thus have size *S* × 1 and are known as *latent variables*. CCA and PLS find the weights that maximize Pearson’s correlation (for the former) or covariance (for the latter) between the brain and behavioural latent variables, thus unravelling low-dimensional brain/behaviour associations. Multiple overlapping associations can be extracted, at most equal to the number of measures available in the smaller dataset; i.e., min(*n*_*x*_, *n*_*y*_). All the weight vectors extracted on either the brain or the behaviour side jointly form an orthonormal basis, so that each association thus draws on distinct parts of the input data. Their respective strength (*i.e*., effect size) is typically reported with Pearson’s correlation coefficient for CCA, and with the fraction of explained covariance for PLS. The ones with smallest strength may be negligible and should be discarded, as typically done by assessing the statistical significance of each association.

The terminology of the CCA/PLS elements varies. In CCA, each pair of latent variables is called a *canonical mode*, while a weight vector and a latent variable are referred to as a *canonical vector* and a *canonical variable*/*variate*, respectively. In PLS, they are respectively called *salience* and *score*. One can also retrieve the relative contributions of brain and behavioural measures to the association by quantifying the absolute correlation between the original and the latent variables, yielding so-called *structure coefficients* (in CCA) or *loadings* (in general). The interpretation of brain-behaviour associations is done by inspecting the magnitude of the weights/loadings for the brain and behavioural variables. The weights are adjusted for the within-dataset correlations, thus indicating the relative contribution of each variable to the latent variable.
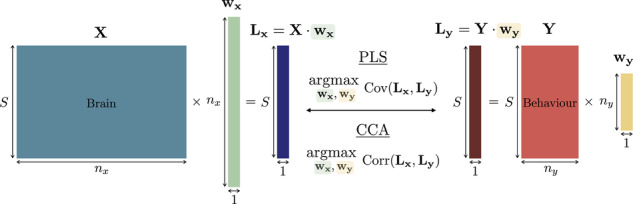

## Brain-behaviour associations in psychiatric disorders

**Fig. 1:**
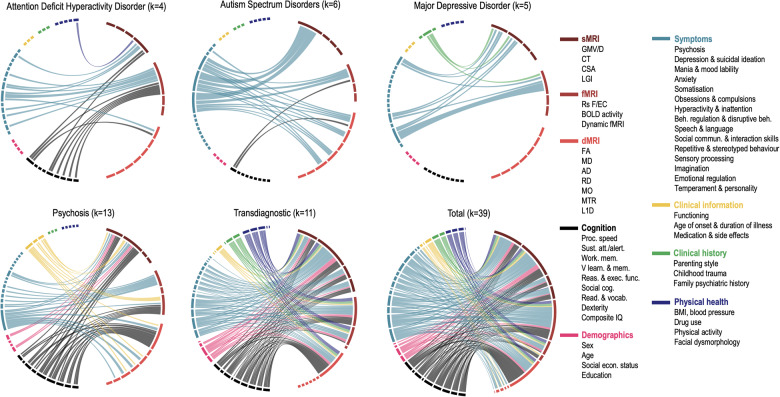
Brain-behaviour associations investigated in the included studies. The size of a connection is proportional to the number of reported models for a given association. Labels are listed clockwise. ADHD: attention deficit hyperactivity disorder, ASD autism spectrum disorders, MDD major depressive disorder, PSD psychosis spectrum disorders, TD transdiagnostic group, sMRI structural MRI, GMV/D grey matter volume/density, CT cortical thickness, CSA cortical surface area, LGI local gyrification indices, fMRI functional MRI, rsFC resting-state functional connectivity, BOLD blood-oxygenation-level-dependent signal, dMRI diffusion MRI, FA fractional anisotropy, MD mean diffusivity, AD axial diffusivity; RD radial diffusivity; MO mode of anisotropy, MTR magnetization transfer ratio; L1D L1 diffusivity.

**Fig. 2:**
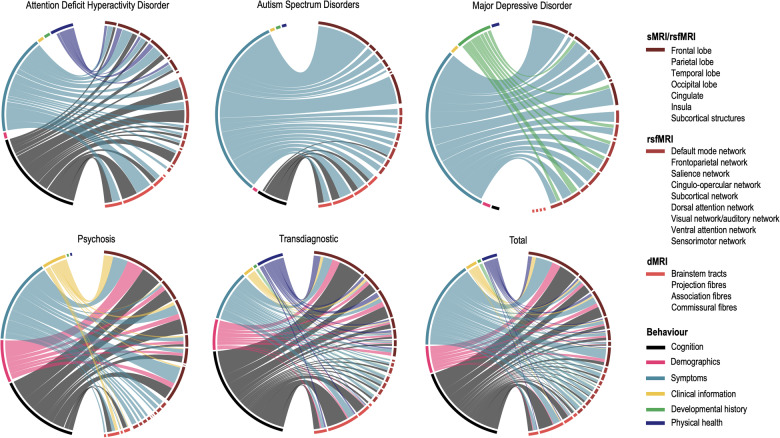
Frequency of brain-behaviour associations for each diagnosis and all diagnoses combined. The thickness of the connections is proportional to the number of reported associations within each diagnostic group. Labels are listed clockwise.

Our systematic review identified 39 studies that investigated brain-behaviour associations in mental health disorders using CCA/PLS (see supplementary methods for search strategy and data extraction) across four diagnostic groups: four in attention deficit and hyperactivity disorder (ADHD, N = 569), six in autism spectrum disorders (ASD, N = 1731), five in major depressive disorder (MDD; N = 938) and thirteen in psychosis, including three in individuals at clinical high-risk (CHR-P, N = 278), five in first episode (FEP, N = 455) and five in established schizophrenia (SZ, N = 417). An additional eleven studies were carried out in large samples including several diagnoses (Transdiagnostic; N = 5731) including SZ, bipolar disorder, ADHD and ASD. Twenty-six studies (67%) used CCA (or regularised CCA). The main characteristics of the included studies are summarised in Tables S2–S6. Figure 1 shows the brain-behaviour associations that were investigated for each diagnostic group and total sample, while Fig. 2 displays, for the same groups, the frequency of statistically significant associations. Overall, studies focused mostly on the association between either brain morphology, resting-state functional connectivity or fractional anisotropy against symptoms and/or cognition. A wide range of additional behavioural domains were also investigated, namely demographics, clinical information and history as well as physical health (Fig. 1).

Behaviour was associated with widespread regions, networks and white matter tracts across the brain for all diagnostic groups. In ADHD, cognition and symptoms were mostly linked with the default mode and frontoparietal networks and white matter association fibres, whereas in ASD, symptoms were mostly associated with the morphology of frontal and subcortical regions, as well as white matter association fibres. Similarly, depressive symptoms in MDD were also associated mostly with frontal regions but also with limbic regions and the cingulate cortex. In psychosis, all types of behavioural features were more visibly associated with frontal regions, followed by temporal and subcortical regions. Finally, the prevalence of frontal regions was also clear in the transdiagnostic group, especially in the association with cognition; white matter association fibres were also linked with cognition and symptoms. Notably, there was no clear association between specific patterns of functional connectivity and behaviour (Fig. 2). In the next sections, the brain-behaviour associations for each diagnostic group are presented in more detail.

### Attention deficit and hyperactivity disorder

Four studies investigated the association between ADHD symptoms/ general cognition and either cortical gyrification [58], resting-state functional connectivity [59, 60] or fractional anisotropy [61]. The former also included measurements of facial dysmorphology typically associated with pre-natal alcohol exposure alongside full scale IQ, behavioural, emotional and cognitive regulation, and inattention and hyperactivity/impulsivity scores. Results showed one significant latent variable, in which lower gyrification in prefrontal, insular, cingulate, temporal, and parietal cortices was associated with greater facial dysmorphia and hyperactivity/impulsivity, as well as poorer IQ and behavioural regulation [58]. Lin et al. [59] found one significant canonical mode where higher hyperactivity/impulsivity and lower cognitive function were associated with higher connectivity predominantly between the default mode, frontoparietal, cingulo-opercular (*e.g*., insula and thalamus) and subcortical (*e.g*., pallidum and putamen) networks. Luo et al. [60] also found several canonical links between several cognitive domains, ADHD and other symptoms (*e.g*., depression, anxiety, social difficulties) with the default mode and frontoparietal networks. Finally, Tsai et al. [61] reported one significant canonical mode, where higher white matter microstructural integrity in multiple brain systems, mostly in association fibres (*e.g*., cingulum, inferior fronto-occipital, uncinate and longitudinal fasciculi), was negatively associated with attention dysregulation, aggression, anxiety, depressive and ADHD symptoms, and positively associated with cognitive function.

### Autism spectrum disorders

The only three studies mostly investigated the association between different ASD symptoms including difficulty in social interaction and communication, stereotyped behaviour and sensory processing against brain morphology [62, 63], functional connectivity [64, 65] and white matter integrity [66, 67]. In a large sample of 325 participants, Mei et al. [62] found one significant canonical mode, in which stronger negative weights on social communication difficulties and stereotyped behaviour were associated with higher weights on grey matter volume in the lateral occipital and superior parietal lobes, right precentral gyrus, amygdala, hippocampus and parahippocampal gyrus and positively with grey matter volume in the thalamus and putamen. In a separate CCA, the same study also found that higher frequency of sensory symptoms was associated with less volume in the cerebellum, lateral occipital and parietal lobes, precentral and inferior frontal gyri and middle frontal lobe. Using the same sample, Mei et al. [66] expanded on these findings by combining grey matter volume and white matter microstructure as input features along with core ASD symptoms. The only significant canonical mode was dominated by white matter features in the inferior and superior longitudinal fasciculus, inferior fronto-occipital fasciculus, corticospinal tract and thalamic radiation, which was associated with repetitive and stereotyped behaviour. Similar tracts, including the inferior longitudinal and uncinate fasciculi, corpus callosum, corticospinal and frontal aslant tracts, thalamic radiation and cingulum were also associated with affect, behavioural, and cognitive dysregulation in Ni et al. [67]. Another study attempted to parse brain-behaviour heterogeneity and found four subtypes in which social difficulties correlated mostly with either fronto-parietal regions, brainstem, temporal-occipital or fronto-subcortical regions [63]. As for functional connectivity, repetitive and stereotyped behaviour as well as social difficulties were associated mainly with the visual network [64, 65].

### Major depressive disorder

Overall, four studies covered a wide range of behavioural features, namely depressive and anxiety symptoms, personality traits and childhood trauma, in association with either brain morphology or resting-state functional connectivity. Drysdale et al. [68] found two clinical profiles – anhedonia & psychomotor retardation and anxiety & insomnia – associated with different functional connectivity networks. The former profile was mostly associated with subcortical structures, as well as the default mode and salience networks, whereas the latter was associated almost exclusively with subcortical regions, especially limbic structures. A similar study with a more comprehensive set of behavioural features (anxiety, depression, personality traits, childhood trauma) found a significant association between functional connectivity in similar networks, especially between subcortical structures, the dorsal attention, sensorimotor, ventral attention networks and childhood trauma [69]. When investigating the association of the same behavioural features with brain morphology, anxious misery was negatively associated with grey matter volume in several cortical and subcortical structures including the middle cingulate and frontal gyri, frontal pole, bilateral amygdala, right hippocampal and superior frontal gyri [70]. Conversely, positive personality traits were positively associated with grey matter volume in the central operculum, posterior and middle cingulate gyri, hippocampus, and amygdala, whereas childhood trauma was negatively associated with grey matter volume in the entorhinal cortex and left hippocampal gyrus. Finally, somatisation was associated with functional connectivity in a widespread group of brain regions including the occipital, temporal and frontal lobes as well as the cerebellum.

### Psychosis

Thirteen studies investigated the association between either brain morphology, resting-state connectivity or FA against a wide range of cognitive and clinical measures including, amongst others, symptoms, functioning, duration of untreated illness and antipsychotic medication. Three studies did not find a statistically significant brain-behaviour association [71–73]. Of the remaining studies, frontal brain regions were more prominently involved in all behavioural domains investigated, followed by temporal and subcortical regions. Symptoms were associated with a wide range of connectivity networks, but mostly with the default mode and sensorimotor networks. As for FA, links between association fibres and cognition, symptoms and clinical information were also observed. In CHR-P, prodromal symptoms and poor functioning were associated with white matter microstructure in association fibres (sagittal stratum, uncinate fasciculus and cingulum) and in the anterior corona radiata [74]. Conversely, cognitive abilities including processing speed, working memory and executive functioning were mostly associated with association fibres (fornix and uncinate fasciculus) and brainstem tracts (superior cerebellar peduncle and medial lemniscus) [75]. In FEP, the association between several behavioural features (*e.g*., cognition, symptom severity, medication, education) and cortical thickness and hippocampal white matter revealed two sex-specific latent variables. In males, greater verbal memory impairments, fewer years of education, greater antipsychotic medication and positive/negative symptomatology were associated with reduced volumes in limbic structures, reduced thickness in mostly parietal and temporal regions, and higher thickness in several frontal regions and in the precuneus. In females, fewer depressive/negative symptoms and an earlier age of onset were associated with reduced volumes in limbic structures, low thickness in temporal and frontal regions and in the cingulate cortex, as well as higher thickness in parietal and occipital regions. In established SZ, cognitive [76, 77] and negative [76] symptoms were associated with grey matter volume in a widespread network of predominately prefrontal, temporal, parietal and (para)limbic regions. A significant association was also found between psychotic symptoms and more time in states characterised by inactive default mode and executive networks, and with heightened activity in the sensorimotor network [78].

### Transdiagnostic groups

Several studies opted for combining diagnostic groups to identify transdiagnostic biomarkers. Half of the studies focused on psychosis spectrum disorders, namely schizophrenia, schizoaffective disorder and bipolar disorder (with and without psychotic features). The remaining studies investigated mostly a combination of ADHD, ASD as well as mood, anxiety, and borderline personality disorder. The majority of studies investigated cognition, which was mainly associated with the morphology of frontal regions. For example, the superior, inferior, caudal and middle frontal cortices were amongst the features with highest loadings across several brain morphology features, namely grey matter volume, cortical thickness, cortical surface area and local gyrification index in the psychosis spectrum group, ADHD, mood and anxiety disorders [79, 80]. As for white matter microstructure, cognition was mostly associated with fractional anisotropy in the superior longitudinal fasciculus, sagittal stratum and fornix [81, 82]. Interestingly, in the only two studies with physical measures amongst a wide range of behavioural features [41, 80], variables such as BMI, drug use and physical activity had the highest loadings across brain modalities, namely morphology, white matter microstructure and functional activity, as well as across brain regions. Overall, psychopathology (*e.g*., psychosis, anxiety) was associated with a wide range of brain modalities and regions, as expected due to the different diagnostic groups. However, only five studies investigated the association between brain and symptoms, all with different diagnoses and/or brain features, making it difficult to ascertain which associations were more prevalent in the different diagnostic groups/symptoms. Notably, three out of the four functional connectivity studies did not find any statistically significant brain-behaviour association [41, 83, 84]; however, they were also amongst the most robust ones due to their rigorous out-of-sample testing.

### Risk of bias

**Fig. 3:**
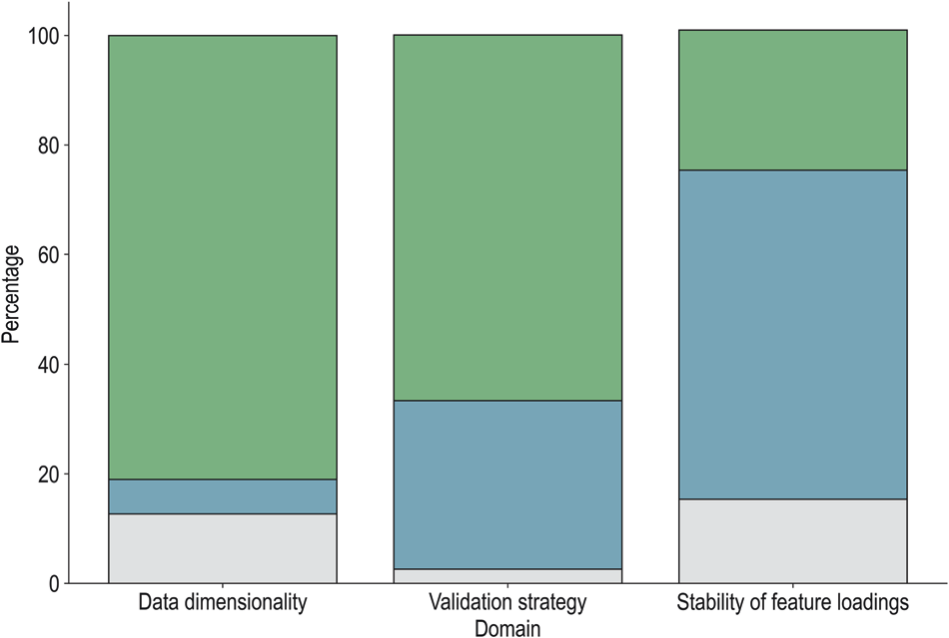
Source of bias in the included studies. Proportion of reported CCA/PLS models with low (blue), high (green) or unclear (grey) bias for the three domains: Data dimensionality, Validation strategy and Stability of feature loadings.

To our knowledge, there are no established tools to systematically assess the risk of bias in ‘doubly’ multivariate brain-behaviour studies. A subjective judgment was made for each study based on three domains that have been consistently highlighted in brain-behaviour studies using these methods: 1) Data dimensionality, 2) Validation strategy, 3) Stability of feature weights. The former was operationalised as the ratio between sample size (N) and total number of brain and behaviour features entered into the CCA/PLS model (*i.e*., effective number of variables, f); an N/f ratio lower than 10 was considered high risk bias [34]. In terms of validation strategy, in-sample testing only and out-of-sample testing (through resampling or an independent dataset) were considered high and low risk of bias, respectively [85]. Finally, studies that did not investigate the stability of feature loadings were rated as high bias for the last domain [85]. If any of the information needed to rate the domains was unclear or missing, the study was rated as ‘unclear’. Most studies (80%) were at high risk of bias for Data dimensionality and 68% were also at high risk due to an in-sample Validation strategy (Fig. 3, supplementary information).

### Generalisability and stability of brain-behaviour associations

**Fig. 4:**
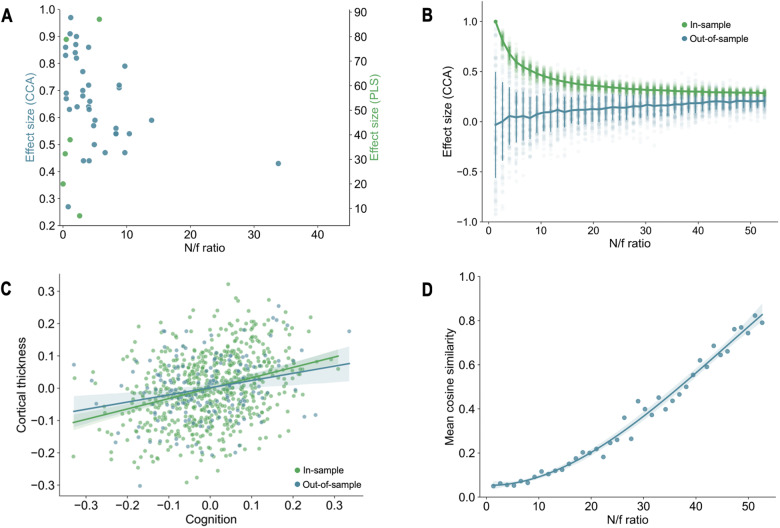
Impact of N/f ratio on model performance and stability. **A** Association between sample size (N) /effective features (f) ratio and effect size (canonical correlation and explained variance for CCA and PLS, respectively) for the first pair of latent variables in the included studies. **B** Impact of sample size on in- and out-of-sample effect size in a CCA between cortical thickness of 68 brain regions (reduced to 5 components with principal component analysis) and 11 cognitive tests in the HCP dataset. The CCA was trained and tested iteratively in 100 random subsamples of the same size for increasing sample sizes (x-axis). The first canonical correlation was used to measure effect size (y axis). The green (in-sample) and blue (out-of-sample) lines show the mean first canonical correlation for each sample size and corresponding standard deviation. **C** First canonical correlation for the total sample (*N* = 842) for in- and out- of sample. **D** Effect of sample size on weight stability. At each sample size, the mean cosine similarity was calculated between the weights of the 100 iterations.

The majority of included studies used a descriptive approach to investigate brain-behaviour associations, that is, CCA/PLS models were trained and tested in the same sample. It is not possible to know whether the same effect size would have held (generalisability) and/or whether the weights would have remained similar (stability) when tested in new data. This is further exacerbated by the fact that most studies had small sample sizes relative to the total number of brain-behaviour features. Although many studies used feature selection/dimensionality reduction to address this, it may not have been enough to mitigate the risk of overfitting as most of them still did not meet the recommended guidelines of 10 participants/feature minimum [34]. More stringent thresholds have been suggested including 42 [86] and 50 [85], indicating that the risk of bias may be even greater. Additionally, very few studies used an independent sample to test their findings. This is a more demanding generalisation than resampling, further highlighting the chances of overfitting. The association between small sample sizes and optimistic performance in predictive modelling is well-established [6, 87]; it can also be observed here (Fig. 4A) and in similar studies in healthy controls [85]. The two limitations, especially when combined, are problematic because CCA and PLS are already prone to overfitting; *i.e*., brain-behaviour associations tend to be much stronger in the sample where the model is trained/fitted (*i.e*., in-sample testing) compared to the associations in a new and unseen sample where the same trained model is applied (*i.e*., out-of-sample testing) [54]. To illustrate how this can lead to biased latent brain-behaviour associations, we present an exemplar standard CCA between cortical thickness (68 original features; 5 after dimensionality reduction) and cognition (11 features) in the Human Connectome Project (HCP) dataset (N = 842, N/f = 52.6) and compare the i) in- and out-of-sample first canonical correlation and ii) weights across models with increasing (*i.e*., more favourable) N/f ratio (Fig. 4B; see supplementary methods). The former investigates how the first (*i.e*., strongest) canonical correlation of a CCA model trained on a subsample of the HCP generalises to another subsample of the same dataset; the latter assesses the stability of model weights; *i.e*., to what extent feature importance varies across multiple models. Ideally, in- and out-of-sample effect sizes should be similar and weights stable across models. However, as expected, for small N/f ratios, there was a clear discrepancy between the means of the in- and out-of-sample effect sizes, and the latter varied substantially, a strong indicator of poor generalizability. As the N/f ratio increased, the effect sizes began to converge, suggesting a more generalizable model. In the total sample, the mean canonical correlation in- and out-of-sample nearly converged at 0.28 (sd=0.02) and 0.21 (sd=0.06), respectively (Fig. 4C). This is in line with more realistic effect sizes for brain-behaviour associations [6]. The stability of the weights (average cosine similarity between feature weights of all 100 iterations for a given N/f ratio; see supplementary methods) follows a similar pattern (Fig. 4D). The weights from models trained in small ratios varied substantially between models (*i.e*., low cosine similarly) suggesting poor stability of feature importance. For the total sample, the cosine similarity was 0.79, indicating a good weight stability. However, the plot suggests that this could be further improved with a larger sample, which is consistent with more recent suggestions that a minimum N/f ratio of 50 is needed for a stable CCA [85]. All combined, these results raise important questions about the validity and reliability of most of the published studies and highlight the need for careful testing to avoid inflated/unstable results. Although some studies used feature selection, dimensionality reduction and/or regularised CCA/PLS, it may not have been enough to mitigate the risk of overfitting. Future studies should follow guidelines for out-of-sample testing, investigating and reporting weights stability and mitigating the effects of high-dimensionality [27, 85, 88].

### Interpretation of brain-behaviour associations in psychiatry

Interpretability of multivariate models is paramount to understand the underlying mechanisms of psychiatric disorders. A strong and robust association which provides no information about its underlying brain and behavioural features would be of limited clinical utility. The interpretation of CCA/PLS models is typically done by inspecting the model’s weights or loadings; the larger the absolute weight/loading, the more a variable contributed to the overall association (Box 1). Importantly, a reliable interpretation of brain-behaviour association requires valid model weights. As shown in Fig. 4D, this may be difficult to secure in small samples. Several studies investigated the stability of weights via bootstrapping and quantified the variability of the weights for each individual variable across iterations. This is a useful strategy as it allows to estimate a confidence interval for each variable weight; however, a more appropriate approach for ’doubly’ multivariate methods would be to report the variability of the entire set of variables’ weights across iterations, using for example cosine similarity. This gives a better estimate of the stability of the entire model’s solution, rather than individual variables. Notably, studies varied in how the most contributing features were identified (*e.g*., top 25% or 5 features, statistically significant features, features with weights above a certain threshold). This is an important limitation of the literature (and by extension, of this review) as it hinders comparisons between studies. In addition, the term ‘brain-behaviour’ should not be confused with any directionality whereby neural features predict behaviour. CCA and PLS-correlation are symmetrical in nature; *i.e*., there is no directionality in covariance and correlation. Although sometimes the terms independent/predictor variables and dependent/criterion variables may be used to describe the brain and behaviour datasets, the order of datasets is ultimately arbitrary [34]. Therefore, the interpretation of associations using these methods should be limited to inferring shared information between brain and behavioural features. Finally, key characteristics of behavioural data and how they can impact the strength and interpretability of associations are less discussed than imaging in brain-behaviour studies [89]. Notably however, CCA/PLS are sensitive to associations both between and within datasets. In practice, this means that small changes in the behavioural data (either due to the choice and/or quality of variables) may markedly alter the composition of the latent variables and ultimately the strength and interpretation of brain-behaviour associations [34]. Some of the included studies focused on symptoms or cognition, while others used different combinations of either/both plus demographics, clinical information and history, and/or physical health. However, the choice of behavioural variable matters; for example, cognition has been shown to have more reliable associations with neural features compared to other behavioural variables such as parent-reported behavioural measures [90]. Similarly, a wider range with less inter-correlated behavioural variables may result in a more diluted brain-behaviour association. Additionally, testing less investigated psychometric properties in psychiatric neuroimaging such as the ability to measure high/low levels and unique/shared variance of the behavioural phenotype (phenotypic resolution and complexity) as well as ability to measure the same construct across different groups (measurement invariance) may help increase the effect size and interpretability of current reliable brain-behaviour associations [89]. Going forward, studies should report key psychometric properties of behavioural measurements (*e.g*., via item-response theory) in their sample in order to increase transparency of the quality of behavioural data. Very few studies have directly investigated the impact of different imaging modalities on brain-behaviour associations [27, 54]. This limited evidence suggests that the behaviour of CCA/PLS is similar between grey matter volume and structural/functional connectivity. However, it is reasonable to expect that modalities/pre-processing pipelines that result in more cohesive (*i.e*., inter-correlated) data across participants will contribute to stronger brain-behaviour associations, much like in behavioural data.

### Prediction of clinical outcomes based on brain-behaviour associations

Most studies used either CCA or PLS as the main approach, that is, the aim of the study was to investigate brain-behaviour associations. Although an interesting aim in itself, these methods can also be used to extract low-dimensional brain-behaviour patterns to be inputted into a machine learning model to predict clinical outcomes, such as relapse or response to treatment; this initial step is known as early fusion [91]. As large brain-behaviour datasets become increasingly accessible, the use CCA/PLS as an early fusion method will likely become more popular. For example, functional connectivity signatures of cognitive performance or symptoms have been used as input for clustering analysis to find subgroups in psychosis [92], ASD [64] and depression [68]. Notably, in a rare effort to replicate findings, the results from the latter study were not reproduced [84]. In the original study, a subset of brain-behaviour features was selected as input for CCA if they were correlated in the entire sample. The replication study addressed this (and other similar limitations) by including the feature selection step and the CCA in the same cross-validation scheme to ensure that both steps were tested for generalisability. Failure to do this is a common source of bias in machine learning studies that leads to optimistic findings [15, 93, 94]. In line with our exemplar CCA application, these two studies highlight the need and the complexity of implementing rigorous out-of-sample testing in brain-behaviour studies. Early fusion could also be used in conjunction with more popular supervised techniques for classification (*e.g*., SVM) or regression (*e.g*., support vector regression) to predict clinical outcomes of interest such as conversion to illness and severity of symptoms. Another possible application is to model brain-behaviour associations in a reference cohort (*e.g*., healthy controls), map patients against this reference model and quantify the deviation from the expected brain-behaviour interaction [95]. Given the known heterogeneity in both neural and behavioural presentations in psychiatric disorders, their interaction is also likely to be heterogeneous [63]. This approach has the advantage of allowing patients to deviate from the expected brain-behaviour interaction in their own unique way, and as such may be a promising approach to model complex and heterogeneous brain-behaviour associations in psychiatry. It would also be interesting to expand on brain-behaviour predictors to other relevant domains such as brain-behaviour-omics associations. This could be achieved via multiset CCA (mCCA), an extension of CCA capable of modelling latent associations between three or more sets of multivariate data [96–98]. Multi-level clustering [99] is another promising approach whereby patients are grouped based on multiple domains, *e.g*., brain and behaviour, simultaneously. For example, Dwyer et al. found a subset of early psychosis patients with low functioning and reduced brain volume [22]. Future studies could investigate how membership of different brain-behaviour subtypes is associated with prognosis. Finally, multi-level networks, where brain and behaviour associations are explored by modelling the links between brain and behaviour networks, are also a promising way forward [100] For example, Hilland et al. developed a joint network of depression-related brain structures and individual depression symptoms [101].

### What to use when

A natural question is which approach should be used: CCA or PLS? Theoretically speaking, the difference lies in the quantity that is being optimized (covariance for PLS *versus* correlation for CCA). In CCA, cross-modality relationships have been normalized by within-block variance; in other words, dependencies between input variables, on either the brain or the behavior side, have been factored out. For this reason, the optimization tends to emphasize unique contributions that variables from the first set make to the prediction of variables within the second. In contrast, collective contributions across different input variables (that is, redundant relationships) are emphasized with PLS [102]. In this review most studies (67%) used CCA; this may be because CCA is an older method that derived from the well-established and popular general linear model, whereas PLS is more recent. Experiments investigating the differences between the two have found that CCA and PLS results are most similar when the correlations within each dataset are low; however, the reliability of CCA worsens with high correlations within either dataset [102]. Notably, PLS has more stable weights in smaller samples [90], however they are more biased and larger samples are needed to have unbiased results compared to CCA [54]. Beyond these findings, knowledge is limited regarding whether other characteristics of the data influence the choice of method. Moreover, given that the stability and reliability of both methods varies with type of input data (*e.g*., parent-reported child behaviour versus cognitive data) [90], each method should be thoroughly investigated for each problem in order to make an informed decision. This should include the strength of within- and between-datasets correlation, testing different numbers of input features (*i.e*., does dimensionality reduction/feature selection improve reliability and stability), in- and out-of-sample associations (*i.e*., quantify the degree of overfitting) and stability of model weights/loadings (*i.e*., using resampling techniques).

### Limitations

This review focused on two popular methods – CCA and PLS – to investigate brain-behaviour associations. Other methods can also be used such as multi-level clustering or independent component analysis. However, application of these methods in brain-behaviour studies in psychiatric disorders is still sparse. In our exemplar CCA, we discuss the importance of ‘out-of-sample’ testing using one large sample. Although this is a significant improvement from in-sample testing, the sub-samples used to find the brain-behaviour association and test it for generalizability follow the same recruitment and assessment procedures, which may mask the true strength of the association. Ideally, testing the CCA model in an independent sample would have yielded a result closer to the true association. Finally, this systematic review was not pre-registered; however, the search strategy is shown in the supplementary information.

## Conclusion

‘Doubly multivariate’ methods, namely CCA and PLS, are becoming a popular approach to investigate brain-behaviour associations in psychiatry. These methods are attractive because they honour the multivariate nature of both brain and behaviour in health and disease. We found 37 studies that used either CCA or PLS to investigate brain-behaviour associations across several diagnostic groups. Overall, our findings suggest that frontal regions may be a transdiagnostic marker for psychopathology and cognition in psychiatric disorders. Although only a small number of studies investigated physical measures such as BMI, and developmental history such as childhood trauma, evidence suggests that these factors may be associated with specific brain signatures. This highlights the importance of expanding the investigation of brain-behaviour studies to include transdiagnostic factors of psychiatric illness in addition to symptoms and cognition. In addition, the choice and quality of behavioural measures should be scrutinized to similar standards as neuroimaging data; this could be achieved, for example, by reporting key psychometric properties of the behavioural data, such as validity, reliability and measurement invariance. Finally, we speculate that the overall effect sizes of the ‘doubly multivariate’ brain-behaviour associations reported so far may be optimistic. This is mainly due to the lack of rigorous out-of-sample testing and the suboptimal ratio between sample size and number of features, which combined may lead to overfitting. In conclusion, the capacity of ‘doubly multivariate’ models to learn complex associations, makes this a promising approach for investigating how brain and behaviour interact in psychiatric disorders. While there are still important challenges to overcome, the findings reviewed here provide preliminary evidence for the potential role of these methods in the investigation of mental health disorders.

### Supplementary information

Prisma_checklist

Supplementary materials
