# Supplementary material for: Multivariate brain-behaviour associations in psychiatric disorders
Source: Transl Psychiatry. 2024 Jun 1;14:231. doi: 10.1038/s41398-024-02954-4 (PMC11144193; doi:10.1038/s41398-024-02954-4)
Supplement: Supplementary file 2 — Supplementary materials [file 41398_2024_2954_MOESM2_ESM.docx]

**Multivariate brain-behaviour associations in psychiatric disorders**

**Supplementary materials**

Table of Contents

[1. Methods 1](#_Toc158891803)

[1.1. Search and Selection Strategy 1](#_Toc158891804)

[1.2. Data Extraction 2](#_Toc158891805)

[2. Results 3](#_Toc158891806)

[2.1. Prisma flowchart 3](#_Toc158891807)

[2.2. Main characteristics of included studies. 4](#_Toc158891808)

[2.3. Sources of bias. 16](#_Toc158891809)

[3. Exemplar Canonical correlation analysis (CCA) 17](#_Toc158891810)

[References 19](#_Toc158891811)

# 1. Methods

## 1.1. Search and Selection Strategy

The electronic database PubMed was searched for relevant publications from inception up to February 2024 to identify studies that used either canonical correlation analysis (CCA) or partial least squares (PLS) to investigate brain-behaviour associations in any psychiatric disorder. Search terms included, but were not limited to, combinations of the following: neuroimaging, and cognition or symptoms or functioning or brain-behaviour, and multivariate or data fusion or canonical correlation analysis or partial least squares (see below for complete search terms). The resulting studies were included 1) if they used CCA, PLS or a variation of the two, 2) to investigate the association between multivariate whole-brain neuroimaging and multivariate behavioural data, 3) in a cross-sectional design, 4) in any psychiatric disorder. Studies were excluded if: i) they did not meet, did not report, or did not sufficiently describe at least one of these inclusion criteria; ii) healthy controls and patients were analysed together but the difference between the two was not reported (except when the CCA//PLS model was not statistically significant/too unstable to investigate meaningful group differences), iii) the brain and behavioural features with highest loadings were not reported, iv) it was the only study for a given psychiatric illness (*i.e.*, required minimum of two studies per diagnostic group). Reference lists of identified articles and relevant review articles were manually screened for additional articles. The results of the literature search are presented in a flow-chart following the PRISMA guidelines ^1^ (Figure S1).

Search terms: ((("neuroimaging"[tw] OR "magnetic resonance imaging"[tw] OR "diffusion tensor imaging"[tw] OR "Positron emission tomography"[tw]) AND (cognition[tw] OR neurocognition[tw] OR symptoms[tw] OR functioning[tw])) OR ("brain-cognition"[tw] OR "brain-behavior"[tw])) AND ("multimodal"[tw] OR multivariate[tw] OR "data fusion"[tw] OR "latent association"[tw] OR "latent variables"[tw] OR "canonical correlation analysis"[tw] OR "partial least squares"[tw])

## 1.2. Data Extraction

The main outcomes of interest were: i) the effect size (*e.g.*, percentage of explained covariance for PLS, canonical correlation for CCA) of the association between the brain and behavioural features sets and ii) the most contributing features for the effect size (*i.e.*, variables with highest absolute weights or correlation between original and latent variables). The following information was also extracted: authors, year of publication, sample size, diagnosis, neuroimaging features, behavioural features, dimensionality, effect size of brain-behaviour association, most contributing features as well as the validation strategy for the effect size and features weights. For studies that tested for the effect of diagnosis (as part of the multivariate model or in *post-hoc* analyses), if it was statistically non-significant, the study was marked as non-significant. There is no standardised way of identifying and reporting the most contributing features for a given effect size in ‘doubly’ multivariate analysis. This information was extracted based on what each study reported as the features with the highest absolute weights. Whenever possible, the top 25% of features were extracted for consistency, as this was the most common method across papers. For reporting and visualisation purposes, the input features and most contributing features were recoded. Behavioural data was grouped into six categories: cognition, demographics, symptoms, family and developmental history, physical health (Table S1).

**Table S1.** Categories for behavioural variables.

| Cognition | Processing speed; Sustained attention/alertness; Working memory; Verbal/visual learning & memory; Reasoning & executive functioning; Social cognition; Reading & vocabulary; Dexterity; Full scale/verbal/performance IQ or another composite score |
| --- | --- |
| Demographics | Sex; Age; Social economic status; Education |
| Symptoms | Psychosis; Depression; Mania & mood lability; Anxiety; Somatisation; Obsessions & compulsions; Hyperactivity & inattention; Behavioural regulation; Speech & Language, Social communication & interaction skills; Repetitive & stereotyped behaviours; Sensory processing; Imagination; Emotional Regulation; Temperament & Personality |
| Clinical information | Functioning; Age onset & duration of illness; Medication & side effects |
| Developmental history | Parenting style; Childhood trauma & negative life events; Family psychiatric history |
| Physical health | Psychical measures (BMI, blood pressure, weight, height, heart rate); Drug use; Physical activity; Facial dysmorphology |

Brain features were mapped onto a well-established atlas for the corresponding modality as much as possible: 1) Desikan atlas ^2^ for voxel- or region-level grey matter volume/density/cortical thickness/surface area/gyrification and region-of-interest fMRI BOLD activity; 2) large-scale functional connectivity networks established in Mohr *et al*. ^3^ for functional connectivity; 3) the ICBM DTI-81 atlas ^4^ for white matter microstructure (*e.g.*, FA). When mapping was not possible, the original feature was used in tables S2-S6 and corresponding high-level structure (*e.g.*, association fibre) in Figure 2. Due to the data-driven and exploratory nature of these methods, several studies tested multiple models. To address this, we extracted only the main result(s); *i.e.*, model(s) that directly answered the main research question or was/were presented as the primary finding(s), as in previous reviews ^5^. Data extraction was performed by two authors separately (SV, MS) and disagreements were discussed to reach a consensus.

# 2. Results

## 2.1. Prisma flowchart

**Figure S1.** Prisma flowchart.

## 2.2. Main characteristics of included studies.

**Table S2.** Attention deficit and hyperactivity disorder.

| **Author, year** | **N** | **Brain features** | **Behaviour features** | **FS/DR** | **Number of effective features (brain/ behaviour)** | **N/F^1^** | **‘Doubly’ multivariate method** | **ES^2^** | **Most contributing features - brain** | **Most contributing features - behaviour** | **Weights stability** |
| --- | --- | --- | --- | --- | --- | --- | --- | --- | --- | --- | --- |
| Kilpatrick 2021 | 62 | LGI | FSIQ; Reasoning, problem solving & executive functioning; Hyperactivity, impulsivity, inattention; Behavioural problems; Emotional regulation; Facial dysmorphology | No | nr/10 | NA | PLS | 59.3% | Precuneus, parahippocampal gyrus, insula, lateral occipital gyrus, superior frontal gyrus, temporo-parieto-occipital junction, secondary visual cortex, supramarginal gyrus | Facial dysmorphology; FSIQ; Behavioural problems; Hyperactivity, impulsivity, inattention | Bootstrapping |
| Lin 2018 | 203 | rsFC | FSIQ; Hyperactivity, impulsivity, inattention | Yes | 3/3 | 33.8 | CCA | 0.43 | Default mode network, frontoparietal network, salience network, cingulo-opercular network, subcortical network | FSIQ; Hyperactivity, impulsivity, inattention | In-sample |
| Tsai 2021 | 182 | FA | FSIQ; Hyperactivity, impulsivity, inattention; Emotional regulation | Yes | 40/5 | 4.0 | CCA | 0.64 | Body of corpus callosum, sagittal stratum, splenium of corpus callosum, cingulum (hippocampus), fornix (cres)/stria terminalis, superior longitudinal fasciculus, uncinate fasciculus, insula, arcuate fasciculus, thalamic radiation of auditory nerve, thalamic radiation of precentral gyrus, posterior commissure | Emotional regulation; FSIQ | In-sample |
| Luo 2023 | 122 | rsFC | Depression; Anxiety; Somatisation; Hyperactivity, impulsivity, inattention; Behavioural problems; Social interaction & communication | Yes | 256/16 | 0.45 | RCCA | 0.83 | Default mode network, sensorimotor network, frontoparietal network | Hyperactivity, impulsivity, inattention | Bootstrapping |
|  |  |  |  |  |  |  |  | 0.81 | Default mode network, visual network, frontoparietal network | Depression; Anxiety; Somatisation |  |
|  |  |  | Processing speed; Attention, vigilance, cognitive control; Working memory; Verbal/visual learning & memory; Reasoning, problem solving & executive functioning | Yes | 256/24 | 0.44 | RCCA | 0.86 | Default mode network, visual network, frontoparietal network | Attention, vigilance, cognitive control; Reasoning, problem solving & executive functioning |  |
|  |  |  |  |  |  |  |  | 0.88 | Default mode network, visual network, frontoparietal network | Processing speed; Verbal/visual learning & memory |  |

^1^Ratio N (number of subjects)/number of features (brain+behaviour) inputted to CCA/PLS; ^2^In-sample effect size, unless otherwise stated; FS/DR: feature selection/dimensionality reduction; ES: effect size; LGI: local gyrification index; rsFC: resting-state functional connectivity; FA: Fractional anisotropy; nr: not reported; NA: not applicable; CCA: canonical correlation analysis; PLS: partial least squares; RCCA: regularised canonical correlation analysis; FSIQ: full-scale IQ.

**Table S3.** Autism spectrum disorders.

| **Author year** | **N** | **Brain features** | **Behaviour features** | **FS/ DR** | **Number of effective features (brain/ behaviour)** | **N/F^1^** | **‘Doubly’ multivariate method** | **ES^2^** | **Most contributing features - brain** | **Most contributing features - behaviour** | **Weights stability** |
| --- | --- | --- | --- | --- | --- | --- | --- | --- | --- | --- | --- |
| Mei 2020 | 325 | GMD | Social interaction & communication; Stereotyped behaviour | Yes | 100/5 | 3.1 | CCA | 0.70 | Amygdala, hippocampus, parahippocampal gyrus, thalamus, putamen, lateral occipital gyrus, superior parietal gyrus, precentral gyrus | Stereotyped behaviour | In-sample, cross-validation |
|  | 194 |  | Social interaction & communication; Stereotyped behaviour; Sensory processing |  | 100/3 | 1.9 |  | 0.84 | Cerebellum cortex, lateral occipital gyrus, precentral gyrus, pars triangularis, pars orbitalis, pars opercularis, rostral middle frontal cortex, caudal middle frontal cortex | Social interaction & communication; Stereotyped behaviour; Sensory processing |  |
| Mei 2022 | 183 | FA, MD, MO, RD, L1D, GMV | Social interaction & communication; Stereotyped behaviour; Sensory processing | Yes | 80/8 | 2.1 | CCA | 0.82 | Sagittal stratum, corticospinal tract, anterior corona radiata, superior longitudinal fasciculus | Stereotyped behaviour | In-sample, cross-validation |
| Ni 2020 | 121 | FA | FSIQ; Emotional regulation | No | 20/4 | 5.0 | CCA | 0.59 | Uncinate fasciculus, sagittal stratum, genu of corpus callosum, body of corpus callosum, cingulum (hippocampus), corticospinal tract, medial lemniscus, thalamic radiation of auditory nerve, thalamic radiation of precentral gyrus, posterior commissure, frontal aslant tract, thalamic radiation of postcentral gyrus | FSIQ; Emotional regulation | In-sample |
| Ilioska 2023 | 232 | rsFC | Social interaction & communication; Stereotyped behaviour | Yes | 48/10 | 4.0 | CCA | 0.72  OOS=0.74 | Sensorimotor network, dorsal attention network, ventral attention network, default mode network, visual network, frontoparietal network, subcortical network | Stereotyped behaviour | In-sample, cross-validation |
|  |  |  |  |  |  |  |  | 0.65  OOS=0.68 | Sensorimotor network, dorsal attention network, ventral attention network, default mode network, visual network, subcortical network | Social interaction & communication |  |
|  | 203 |  | Sensory processing |  | 44/6 | 4.1 |  | 0.63  OOS=0.68 | Visual network, sensorimotor network, subcortical network, frontoparietal network, default mode network, ventral attention network | Sensory processing (sensation seeking, visual/auditory sensitivity, movement sensitivity) |  |
| Zhang 2023 | 571 | GMV | Social interaction & communication; Stereotyped behaviour | No | 140/3 | 4.0 | CCA | OOS=0.86 | Superior parietal cortex, inferior parietal cortex, medial orbitofrontal cortex, pars opercularis, pars orbitalis, pars triangularis | Social interaction & communication | Cross-validation |
|  |  |  |  |  |  |  |  | OOS=-0.65 | Brainstem, subcortical structures, pars compacta | Social interaction & communication |  |
|  |  |  |  |  |  |  |  | OOS=-0.76 | Inferior occipital gyrus, inferior temporal gyrus, middle temporal gyrus, fusiform gyrus | Social interaction & communication |  |
|  |  |  |  |  |  |  |  | OOS=-0.70 | Anterior cingulate cortex, pallidum, brainstem, pars opercularis, pars orbitalis, pars triangularis | Social interaction & communication |  |
| Buch 2023 | 299 | rsFC | Verbal IQ; Social interaction & communication, Stereotyped behaviour | Yes | 350/3 | 0.8 | RCCA | OOS=0.27 | Corticothalamic network, visual network, striatal connectivity | Verbal IQ | Cross-validation |
|  |  |  |  |  |  |  |  | OOS=0.18 | Salience network, visual network, striatal connectivity | Social interaction & communication |  |
|  |  |  |  |  |  |  |  | OOS=0.12 | Corticostriatal connectivity, primary motor areas, frontoparietal network | Stereotyped behaviour |  |

^1^Ratio N (number of subjects)/number of features (brain+behaviour) inputted to CCA/PLS; ^2^In-sample effect size, unless otherwise stated; FS/DR: feature selection/dimensionality reduction; ES: effect size; GMD: grey matter density; FA: fractional anisotropy; MD: mean diffusivity; MO: mode of anisotropy; RD: radial diffusivity; L1D: L1 diffusivity; GMV: grey matter volume; rsFC: resting-state functional connectivity; (R)CCA: (regularised) canonical correlation analysis; PLS: partial least squares; OOS: out-of-sample.

**Table S4.** Major depressive disorder.

| **Author year** | **N** | **Brain features** | **Behaviour features** | **FS/ DR** | **Number of effective features (brain/ behaviour)** | **N/F^1^** | **‘Doubly’ multivariate method** | **ES^2^** | **Most contributing features - brain** | **Most contributing features - behaviour** | **Weights stability** |
| --- | --- | --- | --- | --- | --- | --- | --- | --- | --- | --- | --- |
| Drysdale 2017 | 220 | rsFC | Depression | nr | nr | 1.1 | CCA | 0.91 | Default mode network, frontoparietal network, salience network, cingulo-opercular network, dorsal attention network, subcortical network, visual network/auditory network | Depression (anhedonia, psychomotor retardation) | Bootstrapping |
|  |  |  |  |  |  |  |  | 0.95 | Subcortical network, default mode network, cingulo-opercular network, frontoparietal network, ventral attention network, salience network, sensorimotor network, dorsal attention network | Anxiety (anxiety, insomnia) |  |
| Wu 2021 | 29 | rsFC | Depression | No | 421/38 | 0.3 | PLS | 32.3% | Lingual gyrus, middle temporal gyrus, lateral orbitofrontal cortex, superior temporal gyrus, pericalcarine cortex, pars triangularis, hippocampus, lateral occipital cortex, cerebellum cortex | Somatization | Bootstrapping |
| Yu 2019 | 183 | rsFC | Depression; Anxiety; Trauma; Personality traits | Yes | 55/4 | 3.1 | CCA | 0.68 | Dorsal attention network, subcortical network, sensorimotor network, ventral attention network, frontoparietal network, cingulo-opercular network, visual network/auditory network | Trauma (physical and emotional neglect or abuse) | In-sample |
| Dunlop 2024 | 327 | rsFC | Depression | Yes | nr | NA | RCCA | OOS=0.47 | Default mode network, subcortical network | Mood | Cross-validation |
|  |  |  |  |  |  |  |  | OOS=0.25 | Cingulo-opercular network, dorsal attention network, ventral attention network, visual network | Anhedonia |  |
|  |  |  |  |  |  |  |  | OOS=0.23 | Sensorimotor network | Sleep |  |
| Yu 2021 | 178 | CT & Subcortical structures’ GMV | Anxious misery (depression, anxiety, personality) | No | 22/122 | 1.2 | CCA | 0.97 | Caudal anterior cingulate cortex, posterior cingulate cortex, rostral middle frontal cortex, caudal middle frontal cortex, frontal pole, amygdala, hippocampus, entorhinal cortex, parahippocampal gyrus, superior­ frontal gyrus | Depression, anxiety, personality (neuroticism) | In-sample |
|  |  |  | Positive traits (personality) |  | 22/66 | 2.0 |  | 0.87 | Hippocampus, caudal anterior cingulate cortex, posterior cingulate cortex, amygdala, precuneus, pars triangularis, pars orbitalis, pars opercularis | Personality (extra-version, openness, agreeableness, conscientiousness) |  |
|  |  |  | Trauma (physical and emotional abuse/neglect) |  | 22/20 | 4.2 |  | 0.66 | Entorhinal cortex | Trauma (physical and emotional abuse/neglect) |  |
|  |  |  | Trauma (sexual abuse) |  | 22/5 | 6.6 |  | 0.47 | Hippocampus | Trauma (sexual abuse) |  |

^1^Ratio N (number of subjects)/number of features (brain+behaviour) inputted to CCA/PLS; ^2^In-sample effect size, unless otherwise stated; FS/DR: feature selection/dimensionality reduction; ES: effect size; rsFC: resting-state functional connectivity; CT: cortical thickness; GMV: grey matter volume; nr: not reported; NA: not applicable; (R)CCA: (regularised) canonical correlation analysis; PLS: partial least squares; OOS: out-of-sample.

**Table S5.** Psychosis spectrum disorders.

| **Author, year** | **Diag.** | **N** | **Brain features** | **Behaviour features** | **FS/ DR** | **Number of effective features (brain/ behaviour)** | **N/F^1^** | **‘Doubly’ multivariate method** | **ES^2^** | **Most contributing features - brain** | **Most contributing features - behaviour** | **Weights stability** |
| --- | --- | --- | --- | --- | --- | --- | --- | --- | --- | --- | --- | --- |
| Haas 2020 | CHR-P | 68 | CT & GMV | Language/speech (*e.g.*, amount of speech, semantic properties) | No | 84/39 | 0.6 | RCCA | 0.65 | Pars triangularis, rostral anterior cingulate cortex, superior parietal lobule, superior temporal gyrus, frontal pole, temporal pole, thalamus, hippocampus, caudate nucleus, nucleus accumbens, pallidum, putamen, amygdala, ventral diencephalon | Language/speech (sentence length, semantic coherence, syntactic complexity) | In-sample |
|  |  |  | rsFC |  | No | 11/39 | 1.4 |  | 0.63 | Language network, frontoparietal network, salience network, auditory network, default mode network | Language/speech (coherence, syntactic complexity) |  |
| Krakauer 2017 | CHR-P | 45 | FA, AD, RD, MO | Psychosis; Functioning | No | nr/3 | NA | PLS | 31% | Sagittal stratum, anterior corona radiata, uncinate fasciculus, corticospinal tract, cingulum (cingulate gyrus), cingulum (hippocampus), corpus callosum | Psychosis; Functioning | Bootstrapping |
| Kristensen 2019 | CHR-P | 165 | FA | Verbal IQ; Performance IQ; Processing speed; Attention, vigilance, cognitive control; Working memory; Verbal/visual learning & memory; Reasoning, problem solving & executive functioning; Reading, vocabulary | No | 48/16 | 2.6 | PLS | 7% | Fornix, medial lemniscus | Verbal IQ; Processing speed; Working memory; Reasoning, problem solving & executive functioning | Bootstrapping |
|  |  |  |  |  |  |  |  |  | 5% | Uncinate fasciculus, superior cerebellar peduncle | Verbal IQ; Performance IQ; Processing speed; Working memory; Reasoning, problem solving & executive functioning; Attention, vigilance, cognitive control |  |
| Buck 2022 | FEP | 100 | CT & hippocampal white matter | Sex; Education; Verbal/visual learning & memory; Psychosis; Depression; Age of illness onset; Duration of untreated illness; Antipsychotic medication | No | 80/9 | 1.1 | PLS | 38.0% | Fornix, hippocampus, precentral gyrus, inferior temporal gyrus, middle temporal gyrus, caudal middle frontal gyrus, medial orbitofrontal gyrus, precuneus, superior frontal gyrus, pars opercularis, pars triangularis, pars orbitalis | Sex (male); Verbal/visual learning & memory; Psychosis; Antipsychotic medication; Education | Bootstrapping |
|  |  |  |  |  |  |  |  |  | 20.8% | Rostral middle frontal gyrus, fornix, hippocampus, superior temporal gyrus, entorhinal cortex, pars orbitalis, medial orbitofrontal gyrus, cingulate cortex, precentral gyrus, paracentral gyrus, cuneus, lingual gyrus, mammillary bodies, alveus | Sex (female); Depression; Psychosis; Age of illness onset |  |
| Dean 2013 | FEP | 91 | GMD | Social cognition (reaction time) | No | nr/6 | NA | PLS | ns | - | - | - |
|  |  |  |  | Social cognition (accuracy) |  | nr/6 | NA |  | ns | - | - |  |
| Raghava 2021 | FEP | 51 | FA, AD, RD, MO, MTR | Psychosis | No | 5/4 | 5.7 | PLS | ns | - | - | - |
| Thomas 2021 | FEP | 88 | FA | Processing speed; Attention, vigilance, cognitive control; Working memory; Verbal/visual learning & memory; Reasoning, problem solving & executive functioning; Reading, vocabulary | No | 48/14 | 1.4 | PLS | ns | - | - | - |
| Li 2022 | FEP | 125 | rsFC | Psychosis | No | 227/3 | 0.5 | RCCA | 0.67 OOS=0.69 | Sensorimotor network, default mode network | Psychosis | Bootstrapping, cross-validation |
| Kirschner 2020 | SZ | 182 | GMV | Sex; Education; Composite memory score; Reasoning, problem solving & executive functioning; Reading, vocabulary; Psychosis; Extrapyramidal symptoms | No | nr/15 | NA | PLS | 27.5%  IS=36% | Occipital regions, medial parietal cortex, lateral temporal cortex, medial prefrontal cortex, superior frontal gyrus, posterior cingulate cortex, rostral anterior cingulate cortex, hippocampus, caudate, cerebellum cortex | Composite memory score; Reasoning, problem solving & executive functioning; Reading, vocabulary; Psychosis; Education | Bootstrapping, independent sample |
|  |  |  |  |  |  |  |  |  | 15.0%  IS=ns | Superior frontal gyrus, pars triangularis, pars orbitalis, pars opercularis, rostral anterior cingulate cortex, brainstem, parietal regions, striatum | Composite memory score; Reasoning, problem solving & executive functioning; Reading, vocabulary; Psychosis; Education |  |
|  |  |  |  |  |  |  |  |  | 13%  IS=ns | Cuneus, supramarginal gyrus, precentral gyrus, superior frontal gyrus, rostral middle frontal, caudal middle frontal, pars triangularis, pars orbitalis, pars opercularis | Sex (males); Psychosis; Education |  |
| Kottaram 2019 | SZ | 41 | Dynamic rsfMRI | FSIQ; Cognition composite score; Psychosis; Functioning; Duration of illness; Antipsychotic medication | No | 12/8 | 2.1 | CCA | 0.90 | Default mode network, frontoparietal network, sensorimotor network | Psychosis | In-sample |
| Syeda 2022 | SZ | 86 | CV | Working memory; Verbal/visual learning & memory; Reasoning, problem solving & executive functioning; Reading, vocabulary | No | 68/7 | 1.1 | PLS | 78.9%  r_OOS_=0.47 | Superior temporal gyrus, supramarginal gyrus, inferior temporal gyrus, precentral region, middle temporal region | Working memory; Verbal/visual learning & memory; Reasoning, problem solving & executive functioning | Bootstrapping, cross-validation |
|  |  |  |  |  |  |  |  |  | 13.4%  r_OOS_=0.35 | Caudal anterior cingulate cortex, superior temporal gyrus, inferior temporal gyrus, rostral middle frontal gyrus, middle temporal gyrus | Verbal/visual learning & memory; Reasoning, problem solving & executive functioning |  |
| Zarghami 2022 | SZ | 58 | rsEC | Processing speed; Attention, vigilance, cognitive control; Working memory; Verbal/visual learning & memory; Reasoning, problem solving & executive functioning; Social cognition | Yes | 3/3 | 9.7 | CCA | 0.79  OOS=0.47 | Paracentral lobule, parahippocampal gyrus, inferior temporal gyrus | Social cognition; Reasoning, problem solving & executive functioning | In-sample |
| Holmes 2023 | SZ | 50 | rsFC | Psychosis; Depression; Mania & mood lability; Anxiety; Somatisation; Obsessions & compulsions | Yes | 6/10 | 3.1 | CCA | 0.77 | Sensorimotor network, frontoparietal network, ventral attention network, default mode network, dorsal attention network, visual network | Depression; Psychosis; Somatisation | Bootstrapping |

^1^Ratio N (number of subjects)/number of features (brain+behaviour) inputted to CCA/PLS; ^2^In-sample effect size, unless otherwise stated; Diag.: diagnosis group; FS/DR: feature selection/dimensionality reduction; ES: effect size; CHR-P: clinical high-risk for psychosis; FEP: first-episode psychosis; SZ: schizophrenia; CT: cortical thickness; GMV: grey matter volume; rsFC: resting-state functional connectivity; FA: fractional anisotropy; AD: axial diffusivity; RD: radial diffusivity; MO: mode of anisotropy; GMD: grey matter density; MTR: magnetization transfer ratio; CV: cortical volume; rsEC: resting-state effective connectivity; FSIQ: full-scale IQ; nr: not reported; NA: not applicable; (R)CCA: (regularised) canonical correlation analysis; PLS: partial least squares; ns: non-significant; OOS: out-of-sample; IS: Independent sample; r_OOS_: correlation between brain and behaviour latent variables out-of-sample.

**Table S6.** Transdiagnostic studies.

| **Author year** | **Diag.** | **N** | **Brain features** | **Behaviour features** | **FS/ DR** | **Number of effective features (brain/ behaviour)** | **N/F^1^** | **‘Doubly’ multivariate method** | **ES^2^** | **Most contributing features - brain** | **Most contributing features - behaviour** | **Weights stability** |
| --- | --- | --- | --- | --- | --- | --- | --- | --- | --- | --- | --- | --- |
| Ji 2021 | BDP, SAD, SZ | 436 | rsFC | Processing speed; Working memory; Verbal/visual learning & memory; Reasoning, problem solving & executive functioning; Psychosis | Yes | 180/5 | 2.4 | CCA | OOS=ns | - | - | - |
| Moser 2018 | SZ, BP | 177 | CT, SV, FA, BOLD signal, rsFC | Sex; Age; FSIQ; Psychosis; Depression; Mania & mood lability; Anxiety; Trauma; Antipsychotic dosage; BMI; Lifetime and current drug use; Physical activity; Sedentary time; Psychiatric family history | No | 167/18 | 1.0 | CCA | 0.63 | CT: insula, middle temporal gyrus, lateral orbitofrontal cortex, precentral gyrus, pars opercularis, superior temporal gyrus, fusiform gyrus, posterior cingulate cortex, supramarginal cortex, inferior parietal cortex, precuneus, superior frontal gyrus, pars triangularis, inferior temporal gyrus, rostral middle frontal cortex, paracentral cortex, caudal middle frontal cortex, transverse temporal gyrus, middle frontal gyrus, lateral occipital cortex, lingual cortex, temporal pole, rostral anterior cingulate cortex; FA: posterior thalamic radiation | Age; Sex; FSIQ; BMI; Lifetime substance use | In-sample, cross-validation |
|  |  |  | CT |  |  | 64/18 | 2.2 |  | 0.64 | Middle temporal gyrus, insula, fusiform gyrus, lateral orbitofrontal cortex, superior temporal gyrus, precentral gyrus, pars opercularis, posterior cingulate cortex, inferior temporal gyrus, supramarginal cortex | Age; FSIQ; Antipsychotic dosage; BMI; Psychosis |  |
|  |  |  | SV |  |  | 18/18 | 4.9 |  | 0.50 | Thalamus, caudate, pallidum, putamen | Age; Sex; Lifetime cannabis use; Lifetime substance use; Psychosis |  |
|  |  |  | FA |  |  | 38/18 | 3.2 |  | 0.44 | Posterior thalamic radiation, fornix, body of corpus callosum, genu of corpus callosum, fornix (cres)/stria terminalis, superior longitudinal fasciculus, anterior corona radiata | Age; BMI; Sex; FSIQ; Current alcohol use |  |
|  |  |  | BOLD signal |  |  | 25/18 | 4.1 |  | 0.44 | Caudal anterior cingulate cortex, inferior parietal cortex, middle frontal gyrus, inferior occipital gyrus | FSIQ; Age; BMI; Sedentary time; Psychosis |  |
|  |  |  | rsFC |  |  | 21/18 | 4.5 |  | ns | - | - |  |
| Rodrigue 2018 | SZ, BP, SAD | 678 | GMV | Processing speed; Attention, vigilance, cognitive control; Working memory; Verbal/visual learning & memory; Reasoning, problem solving & executive functioning; Reading, vocabulary | No | 68/14 | 8.3 | CCA | 0.54 | Caudal middle frontal cortex, pars orbitalis, superior frontal cortex, frontal pole, pars opercularis, pars triangularis, precentral gyrus, rostral middle frontal cortex, superior parietal cortex, middle temporal cortex, transverse temporal cortex, cuneus, lateral occipital cortex, rostral anterior cingulate cortex | Reading, vocabulary; Reasoning, problem solving & executive functioning; Processing speed; Verbal/visual learning & memory | In-sample, delete-n Jackknife |
|  |  |  |  |  |  |  |  |  | 0.46 | Pars triangularis, lateral orbitofrontal cortex, medial orbitofrontal cortex, precuneus, supramarginal cortex, inferior parietal cortex, inferior temporal cortex, middle temporal gyrus, insula, fusiform gyrus, parahippocampal cortex, lateral occipital cortex, pericalcarine cortex, lingual cortex, isthmus cingulate cortex, caudal middle frontal cortex | Working memory; Processing speed; Attention, vigilance, cognitive control |  |
|  |  |  | CT |  |  |  |  |  | 0.56 | Caudal middle frontal cortex, paracentral lobule, pars orbitalis, superior frontal cortex, pars triangularis, precentral gyrus, rostral middle frontal cortex, frontal pole, postcentral gyrus, precuneus, superior parietal cortex, supramarginal cortex, lateral occipital cortex, rostral anterior cingulate cortex | Reading, vocabulary; Reasoning, problem solving & executive functioning; Working memory; Processing speed |  |
|  |  |  |  |  |  |  |  |  | 0.49 | Paracentral lobule, pars triangularis, superior frontal cortex, caudal middle frontal cortex, lateral orbitofrontal cortex, pars opercularis, pars orbitalis, pars triangularis, precentral gyrus, rostral middle frontal cortex, inferior parietal cortex, supramarginal cortex, bank of superior temporal sulcus, middle temporal gyrus, superior temporal cortex | Processing speed; Attention, vigilance, cognitive control |  |
|  |  |  | CSA |  |  |  |  |  | 0.54 | Caudal middle frontal cortex, paracentral lobule, rostral middle frontal cortex, superior frontal cortex, lateral orbitofrontal cortex, medial orbitofrontal cortex, pars orbitalis, inferior parietal cortex, precuneus, fusiform gyrus, inferior temporal gyrus, middle temporal gyrus, temporal pole, lateral occipital cortex, rostral anterior cingulate cortex | Reading, vocabulary; Reasoning, problem solving & executive functioning; Processing speed; Verbal/visual learning & memory |  |
|  |  |  |  |  |  |  |  |  | 0.46 | Pars orbitalis, precentral gyrus, pars triangularis, superior frontal cortex, Inferior parietal cortex, postcentral gyrus, precuneus, superior parietal cortex, middle temporal gyrus, parahippocampal cortex, fusiform gyrus, lingual cortex, pericalcarine cortex, cuneus, lateral occipital cortex | Working memory; Processing speed |  |
|  |  |  | LGI |  |  |  |  |  | 0.54 | Pars orbitalis, pars triangularis, paracentral lobule, pars opercularis, parahippocampal cortex, insula | Reading, vocabulary; Reasoning, problem solving & executive functioning; Working memory; Processing speed |  |
| Dinga 2019 | MDD, AD | 187 | rsFC | Depression | Yes | 150/17 | 1.1 | CCA | OOS=ns | - | - | - |
| Kebets 2019 | BP, SZ, SZAD, ADHD | 224 | rsFC | Attention, vigilance, cognitive control; Working memory; Verbal/visual learning & memory; Reasoning, problem solving & executive functioning; Reading, vocabulary; Psychosis; Depression; Mania & mood lability; Anxiety; Somatisation; Obsessions & compulsions; Hyperactivity, impulsivity, inattention; Personality | No | 419^2/54 | 0.003 | PLS | 20% | Sensorimotor network, dorsal attention network, visual network, auditory network, salience network, subcortical network | Mania & mood lability; Hyperactivity, impulsivity, inattention; Anxiety; Obsessions & compulsions; Depression; Psychosis; Somatization; Personality (control) | In-sample, bootstrapping |
|  |  |  |  |  |  |  |  |  | 12% | Sensorimotor network, default network, visual network, auditory network, dorsal attentional network, frontoparietal network, salience network | Reading, vocabulary; Processing speed; Verbal/visual learning & memory; Reasoning, problem solving & executive functioning; Working memory |  |
|  |  |  |  |  |  |  |  |  | 8% | Sensorimotor network, visual network, auditory network, dorsal attention network, salience network, default mode network, frontoparietal network, subcortical network | Hyperactivity, impulsivity, inattention; Personality (novelty seeking, harm avoidance, control); Mania & mood lability; Anxiety (social anxiety) |  |
| Tung 2021 | ADHD, ASD | 595 | FA | FSIQ; Verbal IQ; Performance IQ; Processing speed; Attention, vigilance, cognitive control; Working memory; Verbal/visual learning & memory; Reasoning, problem solving & executive functioning | No | 45/12 | 10.4 | CCA | 0.54 | Fornix, cingulum (hippocampus), posterior thalamic radiation, corticospinal tract, superior longitudinal fasciculus, arcuate fasciculus, frontostriatal circuit (motor), thalamocortical radiation (auditory) | Reasoning, problem solving & executive functioning; Attention, vigilance, cognitive control; Processing speed; Verbal/visual learning & memory | In-sample |
|  |  |  |  |  |  |  |  |  | 0.51 | Corticospinal tract, fornix (cres) / stria terminalis, sagittal stratum, cingulum (hippocampus), superior longitudinal fasciculus, cingulum (cingulate gyrus), arcuate fasciculus, thalamocortical radiation (prefrontal), frontostriatal circuit (motor) | Attention, vigilance, cognitive control; Verbal/visual learning & memory; FSIQ; Reasoning, problem solving & executive functioning |  |
|  |  |  |  |  |  |  |  |  | 0.48 | Superior longitudinal fasciculus, fornix (cres)/stria terminalis, fornix, thalamocortical radiation (Auditory), frontal aslant tract, thalamocortical radiation (sensorimotor), frontostriatal circuit (motor) | Verbal/visual learning & memory; FSIQ; Reasoning, problem solving & executive functioning; Processing speed; Attention, vigilance, cognitive control |  |
|  |  |  |  |  |  |  |  |  | 0.44 | Fornix (cres)/stria terminalis, uncinate fasciculus, superior longitudinal fasciculus, posterior thalamic radiation, corpus callosum (temporal), thalamocortical radiation (auditory), arcuate fasciculus | Attention, vigilance, cognitive control; Verbal/visual learning & memory |  |
|  |  |  |  |  |  |  |  |  | 0.43 | Genu of corpus callosum, sagittal stratum, fornix (cres)/stria terminalis, cingulum (hippocampus), posterior thalamic radiation, corpus callosum (parietal), corpus callosum (temporal), corpus callosum (sensorimotor), frontostriatal circuit (motor), thalamocortical radiation (auditory), corpus callosum (sensorimotor) | Verbal IQ; Performance IQ; Reasoning, problem solving & executive functioning; Attention, vigilance, cognitive control |  |
|  |  | 354 |  | Hyperactivity, impulsivity, inattention; Behavioural problems; Social interaction & communication; Stereotyped behaviour; Imagination | No | 45/28 | 4.8 | CCA | 0.57 | Fornix (cres)/stria terminalis, cingulum (cingulate gyrus), uncinate fasciculus, genu of corpus callosum, superior longitudinal fasciculus, frontal aslant tract, perpendicular fasciculus, corpus callosum (temporal), arcuate fasciculus | Social interaction & communication; Imagination |  |
|  |  |  |  |  |  |  |  |  | 0.54 | Sagittal stratum, cingulum (hippocampus), cingulum (cingulate gyrus), uncinate fasciculus, arcuate fasciculus, corpus callosum (temporal), perpendicular fasciculus, thalamocortical radiation (auditory), corpus callosum (sensorimotor) | Social interaction & communication; Imagination |  |
| Chien 2022 | SZ, ADHD, ASD | 738 | FA z-scores | Processing speed; Attention, vigilance, cognitive control; Working memory; Verbal/visual learning & memory; Reasoning, problem solving & executive functioning | No | 45/7 | 14.2 | CCA | ns | - | - | In-sample |
|  |  |  |  | Processing speed; Working memory; Verbal/visual learning & memory; Reasoning, problem solving & executive functioning; Reading, vocabulary | No | 45/8 | 13.9 | CCA | 0.59 | Sagittal stratum, uncinate fasciculus, superior longitudinal fasciculus, fornix, thalamocortical radiation, corpus callosum, frontostriatal circuit | Working memory |  |
| Kebets 2021 | BP, BPD, ADHD | 166 | BOLD signal | Depression; Mania & mood lability | No | nr/3 | NA | PLS | 74% | Orbitofrontal cortex, rostral middle frontal gyrus, rostral anterior cingulate cortex, amygdala, hippocampus, precentral gyrus, insula, occipital regions | Depression; Mania & mood lability | In-sample |
| Calarco 2023 | SZ, SAD, SFD, DD | 308 | FA | Processing speed; Reasoning, problem solving & executive functioning; Attention, vigilance, cognitive control; Working memory; Verbal/visual learning & memory; Social cognition | No | 16/19 | 8.8 | CCA | 0.72  IS=0.72 | Uncinate fasciculus, body of corpus callosum | Processing speed; Attention, vigilance, cognitive control; Verbal/visual learning & memory; Social cognition | In-sample, independent sample |
| Voldsbekk 2023 | ADHD, anxiety, mood, other | 1732 | CT, CSA, CV, LGI | Reading, vocabulary; Dexterity, Age; Socioeconomic status; Height; Weight; Heart rate; Blood pressure; BMI; Handedness; Physical activity; Personality; Depression; Anxiety; Somatisation; Hyperactivity, impulsivity, inattention; Behavioural problems; Speech/language; Social interaction & communication; Stereotyped behaviours; Sensory processing | Yes | nr | NA | CCA | OOS=0.92 | Precentral gyrus, postcentral gyrus, superior parietal cortex, paracentral lobule, precuneus | Age; Weight; Height; Reading, vocabulary | In-sample, independent sample |
|  |  |  |  |  |  |  |  |  | OOS=0.92 | Lateral orbitofrontal cortex, rostral middle frontal cortex | Social interaction & communication; Behavioural problems; Hyperactivity, impulsivity, inattention; Stereotyped behaviours; Personality |  |
| Bashford-Largo 2023 | MDD,  SAD,  GAD,  PTSD,  CD,  ODD,  ADHD | 490 | GMV | Hyperactivity, impulsivity, inattention; Behavioural problems; Social interaction & communication; Emotional regulation | No | 82/4 | 5.7 | PLS | 87.1% | Fusiform gyrus, insula, superior temporal gyrus, middle temporal gyrus, inferior temporal gyrus, temporal pole, medial orbitofrontal cortex, caudal middle frontal cortex, lateral orbitofrontal cortex, bank of superior temporal sulcus | Behavioural problems (conduct) | Bootstrapping |

^1^Ratio N (number of subjects)/number of features (brain+behaviour) inputted to CCA/PLS; ^2^In-sample effect size, unless otherwise stated; Diag.: diagnosis group; FS/DR: feature selection/dimensionality reduction; ES: effect size; BDP: bipolar disorder patients with psychosis; SZAD: schizo-affective disorder; SZ: schizophrenia; BP: bipolar disorder; MDD: major depressive disorder; AD: anxiety disorder; ADHD: attention deficit and hyperactivity disorder; ASD: autism spectrum disorders; BPD: borderline personality disorder; SFD: schizophreniform disorder; DD: delusional disorder; SAD: social anxiety disorder; GAD: generalized anxiety disorder; PTSD: posttraumatic stress disorder; CD: conduct disorder; ODD: oppositional defiant disorder; rsFC: resting-state functional connectivity; CT: cortical thickness; SV: subcortical volume; FA: fractional anisotropy; BOLD: blood oxygenation level-dependent; GMV: grey matter volume; CSA: cortical surface area; LGI: local gyrification index; CV: cortical volume; FSIQ: full-scale IQ; nr: not reported; NA: not applicable; CCA: canonical correlation analysis; PLS: partial least squares; OOS: out-of-sample

## 2.3. Sources of bias.

# 3. Exemplar Canonical correlation analysis (CCA)

Participants from the Human Connectome Project (HCP) 1200 subjects release ^6^ were used in the analysis. Table S7 summarises the 11 cognitive tests and respective cognitive domains and variables used (for a description of the tests see the [HCP reference manual](https://www.humanconnectome.org/storage/app/media/documentation/s1200/HCP_S1200_Release_Reference_Manual.pdf)).

**Table S7.** Cognitive tests from the Human Connectome Project used in the CCA analysis.

| **Cognitive test** | **Cognitive domain** | **Variable(s) used** |
| --- | --- | --- |
| Picture Sequence Memory | Episodic Memory | PicSeq_AgeAdj |
| Dimensional Change Card Sort | Executive Function/Cognitive Flexibility | CardSort_AgeAdj |
| Flanker Task | Executive Function/Inhibition | Flanker_AgeAdj |
| Penn Progressive Matrices | Fluid Intelligence | PMAT24_A_CR |
| Oral Reading Recognition | Language/Reading Decoding | ReadEng_AgeAdj |
| Picture Vocabulary | Language/Vocabulary Comprehension | PicVocab_AgeAdj |
| Pattern Completion Processing Speed | Processing Speed | ProcSpeed_AgeAdj |
| Delay Discounting | Self-regulation/Impulsivity | DDisc_AUC_40K |
| Penn Word Memory Test | Verbal Episodic Memory | IWRD_TOT |
| List Sorting | Working Memory | ListSort_AgeAdj |
| Penn Emotion Recognition Test | Emotion Recognition | ER40_CR |

Structural T1-weighted images were acquired using a magnetization-prepared rapid acquisition gradient-echo (MPRAGE) sequence (TR = 2400 ms, TE = 2.14 ms, flip angle = 8°, FOV = 224 × 224, 0.7 mm isotropic voxels) in a Siemens 3T “Connectome Skyra” scanner. Already pre-processed and quality-controlled images were downloaded from the HCP database (see [HCP reference manual](https://www.humanconnectome.org/storage/app/media/documentation/s1200/HCP_S1200_Release_Reference_Manual.pdf) for more details). The cortical thickness of 68 cortical regions (34 per hemisphere) were extracted based on the Desikan-Killiany atlas ^2^. Both brain and cognitive data were normalised by removing the mean and scaling to unit variance. Normalised data were screened for outliers and participants with z-scores above or below 3 were removed (N=203). The final sample comprised 842 participants (55% female) between 22 and 37 years (Figure S2A). Initial analyses did not show significant deviations from univariate normality or evidence of multicollinearity (Figure S2B and C). Participants were divided into training (80%) and test (20%) sets. Each feature was scaled independently by removing the mean and scaling to unit variance, and principal component analysis (PCA) was used to reduce brain data dimensionality. We tested three values for number of principal components (k=5, k=10 and k=15). The model with 5 principal components was chosen as it showed better CCA stability (measured by cosine similarity across CCA weights; Table S8). The final 11 cognitive features and 5 brain features were entered in a CCA model with default parameters and the first canonical correlation of either set was extracted. Normalization, PCA and CCA were cross-validated; *i.e.*, fitted to the training set first and then applied to the test set. This process was repeated 100 times for a given sample size, each time with a random partition between training and test sets. Sample sizes increased in 40 increments of 2.5% of the total sample each, totalling 4000 models (*i.e.*, 40 times 100 models); subsamples were extracted at random without replacement. Model weights stability was assessed by calculating the mean cosine similarity (similarity between two vectors) between all pairwise weights of the 100 iterations for a given sample size. All data transformations and data analyses were implemented using the scikit-learn library (version 1.1) ^7^ in Python 3.9.


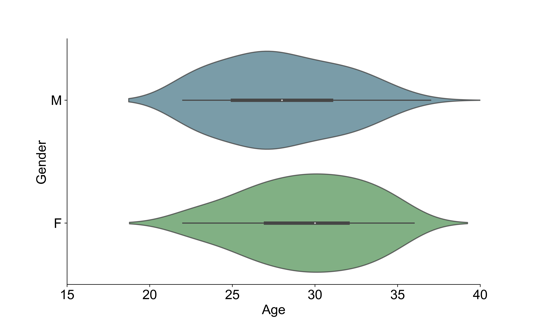

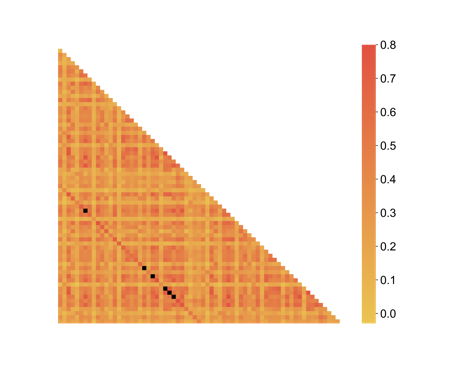


A

B

A

A

B


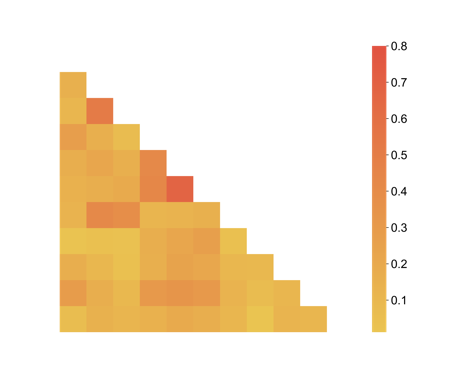


C

C


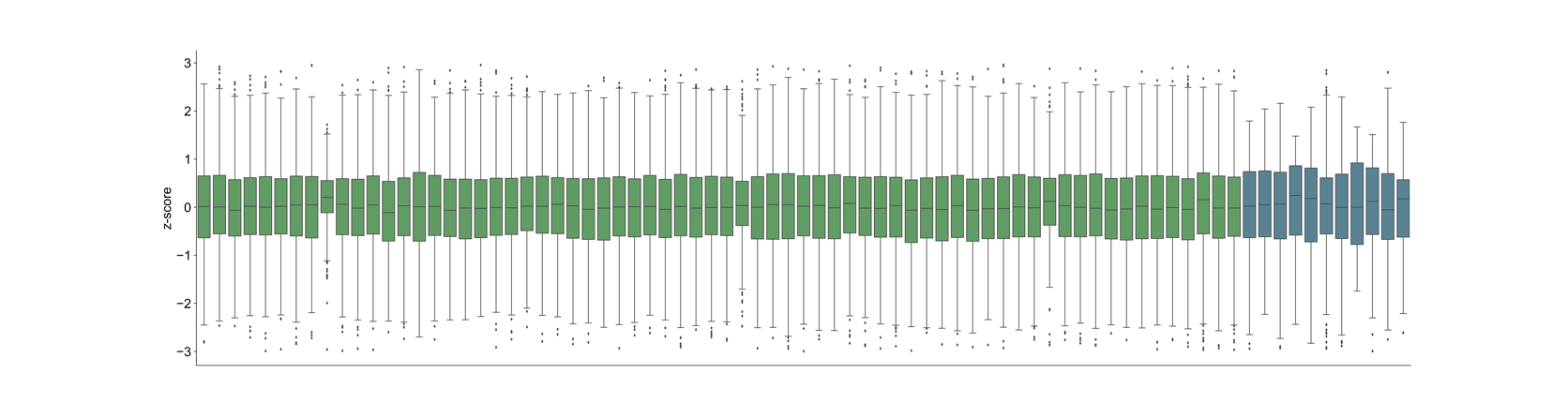


**Figure S2. Main characteristics of the final sample used in CCA.** **(A)** Age distribution for males (M) and females (F). **(B)** Pearson’s correlation matrices between cognitive (left) and brain (right) features; correlations above .80 are shown in black. **(C)** Distributions for brain (green) and cognitive (blue) z-scores.

**Table S8.** Number of principal components and respective canonical correlation in the train and test sets, and cosine similarity for the total sample.

| Number of principal components | CCA 1^st^ canonical correlation – training set | CCA 1^st^ canonical correlation – test set | Cosine similarity |
| --- | --- | --- | --- |
| 5 | 0.28 (0.02) | 0.21 (0.06) | 0.79 |
| 10 | 0.32 (0.01) | 0.22 (0.06) | 0.62 |
| 15 | 0.36 (0.02) | 0.26 (0.07) | 0.41 |

# References

1 Moher D, Liberati A, Tetzlaff J, Altman DG. Preferred reporting items for systematic reviews and meta-analyses: the PRISMA statement. *BMJ* 2009; **339**. doi:10.1136/bmj.b2535.

2 Desikan RS, Ségonne F, Fischl B, Quinn BT, Dickerson BC, Blacker D *et al.* An automated labeling system for subdividing the human cerebral cortex on MRI scans into gyral based regions of interest. *Neuroimage* 2006; **31**: 968–980.

3 Mohr H, Wolfensteller U, Betzel RF, Mišić B, Sporns O, Richiardi J *et al.* Integration and segregation of large-scale brain networks during short-term task automatization. *Nat Commun* 2016; **7**: 13217.

4 Mori S, Oishi K, Jiang H, Jiang L, Li X, Akhter K *et al.* Stereotaxic white matter atlas based on diffusion tensor imaging in an ICBM template. *Neuroimage* 2008; **40**: 570–582.

5 Vieira S, Liang X, Guiomar R, Mechelli A. Can we predict who will benefit from cognitive-behavioural therapy ? A systematic review and meta-analysis of machine learning studies. *Clin Psychol Rev* 2022; **97**: 102193.

6 Glasser MF, Smith SM, Marcus DS, Andersson JLR, Auerbach EJ, Behrens TEJ *et al.* The Human Connectome Project’s neuroimaging approach. *Nat Neurosci* 2016; **19**: 1175–1187.

7 Pedregosa F, Varoquaux G, Gramfort A, Michel V, Thirion B, Grisel O *et al.* Scikit-learn: Machine Learning in Python. *J Mach Learn Res* 2011; **12**: 2825–2830.
